# The C962R ORF of African Swine Fever Strain Georgia Is Non-Essential and Not Required for Virulence in Swine

**DOI:** 10.3390/v12060676

**Published:** 2020-06-23

**Authors:** Elizabeth. Ramirez-Medina, Elizabeth. A. Vuono, Ayushi. Rai, Sarah. Pruitt, Ediane. Silva, Lauro. Velazquez-Salinas, James. Zhu, Manuel. V. Borca, Douglas. P. Gladue

**Affiliations:** 1 Plum Island Animal Disease Center, Agricultural Research Service (ARS), United States Department of Agriculture (USDA), Greenport, NY 11944, USA; elizabeth.ramirez@usda.gov (E.R.-M.); Ayushi.Rai@usda.gov (A.R.); Sarah.Pruitt@usda.gov (S.P.); Ediane.Silva@usda.gov (E.S.); Lauro.Velazquez@usda.gov (L.V.-S.); James.Zhu@usda.gov (J.Z.); 2 Department of Pathobiology and Veterinary Science, University of Connecticut, Storrs, CT 06269, USA; 3 Department of Pathobiology and Population Medicine, Mississippi State University, P.O. Box 6100, Starkville, MS 39762, USA; Elizabeth.Vuono@usda.gov; 4 Oak Ridge Institute for Science and Education (ORISE), Oak Ridge, TN 37830, USA; 5 Department of Anatomy and Physiology, Kansas State University, Manhattan, KS 66506, USA

**Keywords:** ASFV, ASF, African swine fever virus, C962R

## Abstract

African swine fever virus (ASFV) is the causative agent of the African swine fever (ASF) epizootic currently affecting pigs throughout Eurasia, causing significant economic losses in the swine industry. The virus genome encodes for more than 160 genes, of which only a few have been studied in detail. Here we describe the previously uncharacterized ASFV open reading frame (ORF) *C962R*, a gene encoding for a putative NTPase. RNA transcription studies using infected swine macrophages demonstrate that the C962R gene is translated as a late virus protein. A recombinant ASFV lacking the *C962R* gene (ASFV-G-ΔC962R) demonstrates in vivo that the *C962R* gene is non-essential, since ASFV-G-ΔC962R has similar replication kinetics in primary swine macrophage cell cultures when compared to parental highly virulent field isolate Georgia2007 (ASFV-G). Experimental infection of domestic pigs with ASFV-G-ΔC962R produced a clinical disease similar to that caused by the parental ASFV-G, confirming that deletion of the *C962R* gene from the ASFV genome does not impact virulence.

## 1. Introduction

African swine fever virus (ASFV) is the causative agent of a very contagious and frequently lethal viral disease of swine, African swine fever (ASF). The viral infection produces various clinical presentations in domestic pigs, from subclinical to highly lethal, depending on the acting viral strain [1]. Currently, there is no commercial vaccine available for ASF, and therefore, ASF outbreaks are mainly controlled by culling susceptible animals in infected farms. ASF has historically remained endemic in sub-Saharan countries, as well as in Sardinia (Italy). In 2007, ASFV was detected in the Republic of Georgia, quickly spreading to neighboring countries, and is now present in Eastern Europe [2]. Recently, the 2018 introduction of ASFV into China was followed quickly by it spreading throughout mainland Asia, the Philippines and most recently as far south as Timor-Leste and Papua New Guinea, just off the coast of Australia. This continued spread of ASFV has the potential to cause a worldwide protein availability shortage and large economic losses in the swine industry [3]. 

Controlling of ASF relies on detection and culling of infected farms and maintaining strict bio-security measures to prevent the introduction of ASFV. There is no commercial vaccine for ASFV, and all experimental vaccines that show promise to the current circulating strain in Europe and Asia are live attenuated vaccines designed from the circulating isolate that originated in the Republic of Georgia, containing the deletion of one or more proteins from field isolates [4,5,6,7,8,9]. Understanding the role of individual genes and how their possible manipulation could be used to develop experimental vaccines is critical to help develop next generation ASF vaccines. 

ASFV is structurally complex, with a large (180–190 kilobase pairs) double-stranded DNA genome harboring more than 160 open reading frames (ORF) [2]. However, the function of most of these ORFs has not been experimentally characterized, and their role is only based on sequence homology prediction to proteins with known function. In particular, the role of specific ASFV genes involved in viral virulence still remains poorly known [1]. Identifying viral proteins that are important for in vitro and in vivo virus replication, and importantly, in virus virulence in swine, is critical to developing novel countermeasures to control the disease. Discovery of ASFV gene function via genetic manipulation has enabled the production of experimental ASFV live-attenuated vaccine candidates [4,5,8,10,11]. Interestingly, just a small number of virus genes have been successfully deleted from the ASFV genome, producing novel recombinant virus (e.g.,: TK, NL, CD2, MGF360-16R, L83L) [12,13,14,15,16], and another small number of genes determined to be essential for virus replication (e.g.,: EP152R, p30, p54, p72) [17,18,19,20]. The absence of experimental information restricts the knowledge for most ASFV proteins to ORF analysis by functional genomics, predicting the functions of these ORFs.

In this report, we study the role of a previously uncharacterized ASFV gene, *C962R,* a highly conserved protein among ASFV isolates that is similar to the iridovirus primase family fused to D5-like helicase domains [21]. In some older annotations, the primase and helicase domains are annotated separately, but there is no evidence that the primase protein would be separate, as it lacks a stop codon, joining the two domains into one protein. In this study, a recombinant ASFV lacking the non-essential *C962R* gene was constructed (ASFV-G-ΔC962R) and assessed in vitro and in vivo. Experimental infection of domestic pigs with ASFV-G-ΔC962R demonstrated that C962R is not required for virus virulence.

## 2. Materials and Methods 

### 2.1. Viruses and Cells 

Primary swine macrophage cell cultures were prepared from defibrinated swine blood, as previously described in detail [22]. Briefly, heparin-treated swine blood was incubated at 37 °C for 1 h to allow sedimentation of the erythrocyte fraction. Mononuclear leukocytes were separated by flotation over a Ficoll-Paque (Pharmacia, Piscataway, N.J., USA) density gradient (specific gravity, 1.079). The monocyte/macrophage cell fraction was cultured in plastic Primaria (Falcon, Becton Dickinson Labware, Franklin Lakes, N.J., USA) tissue culture flasks containing macrophage media, composed of RPMI 1640 Medium (Life Technologies, Grand Island, NY, USA) with 30% L929 supernatant and 20% fetal bovine serum (HI-FBS, Thermo Scientific, Waltham, MA, USA) for 48 h at 37 °C under 5% CO2.

Adherent cells were detached from the plastic by using 10 mM EDTA in phosphate buffered saline (PBS), and were then reseeded into Primaria T25, 6- or 96-well dishes at a density of 5 × 10^6^ cells per mL for use in assays 24 h later. ASFV Georgia (ASFV-G) was a field isolate kindly provided by Dr. Nino Vepkhvadze from the Laboratory of the Ministry of Agriculture (LMA) in Tbilisi, Republic of Georgia [10]. Comparative growth curves between ASFV-G-ΔC962R and parental ASFV-G were performed in primary swine macrophage cell cultures. Preformed monolayers were prepared in 24-well plates and infected at a MOI of 0.1 (based on HAD_50_ previously determined in primary swine macrophage cell cultures). After 1 h of adsorption at 37 °C under 5% CO2 the inoculum was removed, and the cells were rinsed two times with PBS. The monolayers were then rinsed with macrophage media and incubated for 2, 24, 48, 72 and 96 h at 37 °C under 5% CO2. At appropriate times post-infection, the cells were frozen at ≤−70 °C and the thawed lysates were used to determine titers by HAD_50_/mL in primary swine macrophage cell cultures. All samples were run simultaneously to avoid inter-assay variability. Virus titration was performed on primary swine macrophage cell cultures in 96-well plates. Virus dilutions and cultures were performed using macrophage medium. The presence of virus was assessed by hemadsorption (HA), and virus titers were calculated as previously described [23].

### 2.2. Construction of the Recombinant Viruses 

**Figure 1:**
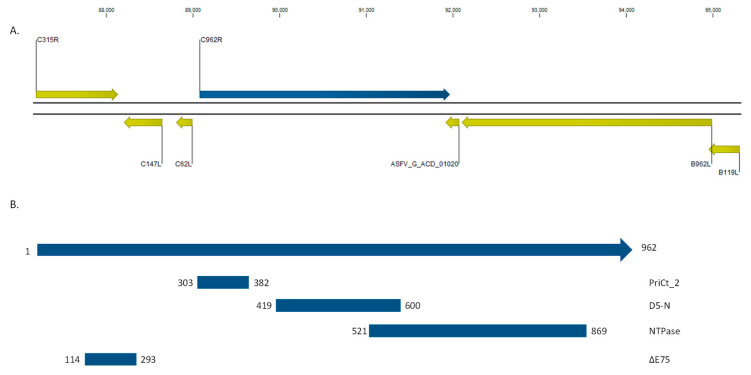
(**A**) Schematic representation of the *C962R* ORF (blue) in the ASFV-G genome, showing adjacent open reading frames (yellow). (**B**) Locations of the predicted functional domains in C962R, positions indicated are amino acid positions indicating the boundaries of the predicted domain. ∆E75 represents the region that is deleted in the ASFV E75 isolate.

Recombinant ASFV-G-ΔC962R was generated by homologous recombination between the parental ASFV genome and a recombination transfer vector following previously described procedures [6]. The recombinant transfer vector (p72mCherryΔC962R) contained flanking genomic regions: the left arm is located between genomic positions 87,875–89,074, and the right arm is located between genomic positions 91,899–93,098 and a reporter gene cassette containing the mCherry fluorescent protein (mCherry) gene under the control of the ASFV p72 late gene promoter [24]. The recombinant transfer vector was obtained by DNA synthesis (Epoch Life Sciences, Sugar Land, TX, USA). This construction created a 2824-nucleotide deletion between nucleotide positions 89,075–91,898, deleting most of the ORF sequence for *C962R* with the coding region for the last 65 nucleotides of the C-terminus remaining, but without a promoter or start codon, making the transcription of this product unlikely (Figure 1). The remaining 65 nucleotides of *C962R* were left in place to avoid altering the transcription of ORF ASFV-G-AVD_01020, which has an overlapping ORF potentially transcribed in the opposite direction of *C962R.* Macrophage cell cultures were infected with ASFV-G and transfected with p72mCherryΔC962R. Recombinant ASFV-G-ΔC962R was purified to homogeneity by successive rounds of limiting dilution purification. ASFV DNA was extracted from infected cells, and full-length sequence using next generation sequencing (NGS) was performed as described previously [24], using an Illumina NextSeq500 sequencer, using standard sequencing protocols. Analysis of the sequence was done using CLC Genomics Workbench software version 20 (QIAGEN, Hilden, Germany).

### 2.3. Animal Experiments

ASFV-G-ΔC962R was assessed for its virulence phenotype relative to the parental ASFV-G virus using 80–90-pound commercial breed swine. Five pigs were inoculated intramuscularly (IM) with 10^2^ HAD_50_ of ASFV-G-ΔC962R and compared with a group of pigs inoculated with similar doses of ASFV-G. Clinical signs (anorexia, depression, fever, purple skin discoloration, staggering gait, diarrhea and cough) and changes in body temperature were recorded daily throughout the experiment. Animal experiments were performed under biosafety level 3 conditions in the animal facilities at Plum Island Animal Disease Center, following a strict protocol approved by the Institutional Animal Care and Use Committee (225.01-16-R_090716).

### 2.4. Ethics Statement

Animal experiments were performed under biosafety level 3AG conditions in the animal facilities at Plum Island Animal Disease Center (PIADC). All experimental procedures were carried out in compliance with the Animal Welfare Act (AWA), the 2011 Guide for Care and Use of Laboratory Animals, the 2002 PHS Policy for the Humane Care and Use of Laboratory Animals, and U.S. Government Principles for Utilization and Care of Vertebrate Animals Used in Testing, Research and Training (IRAC 1985), as well as specific animal protocols reviewed and approved by the PIADC Institutional Animal Care and Use Committee of the U.S. Departments of Agriculture and Homeland Security (protocol number 225.04-16-R, 09-07-16).

## 3. Results and Discussion

### 3.1. C962R Gene Is Conserved across Different ASFV Isolates and Transcribed as a Late Viral Gene

ASFV ORF *C962R* is located on the positive strand of the ASFV Georgia2007 field isolate (ASFV-G) genome between positions 89,075 and 91,963; the C-terminus of the protein overlaps with the C-terminus of protein ACD_01020, predicted to be transcribed on the negative strand of the ASFV genome. InterPro sequence analysis [25] revealed that C962R contains two Pfam domains: one between amino acids 303–382 (PriCT_2), an alpha-helical domain found at the C-terminal of Primases, and a second domain between amino acids 419–600 (D5-N) found in D5 proteins of DNA viruses and bacteriophage P4 DNA Primase. C962R also contains a PROSITE helicase domain (SF3_Helicase_1) spanning amino acids 607–775; this region is further predicted by PANTHER prediction (PTHR35372) to be a P-loop containing nucleoside triphosphate hydrolase between residues 521–869. Furthermore C962R was not detected in the proteome of ASFV viral particles, which would be expected for a DNA replication component in ASFV [26]. These predicted domains and location in the genome are shown in Figure 1A.

**Figure 2:**
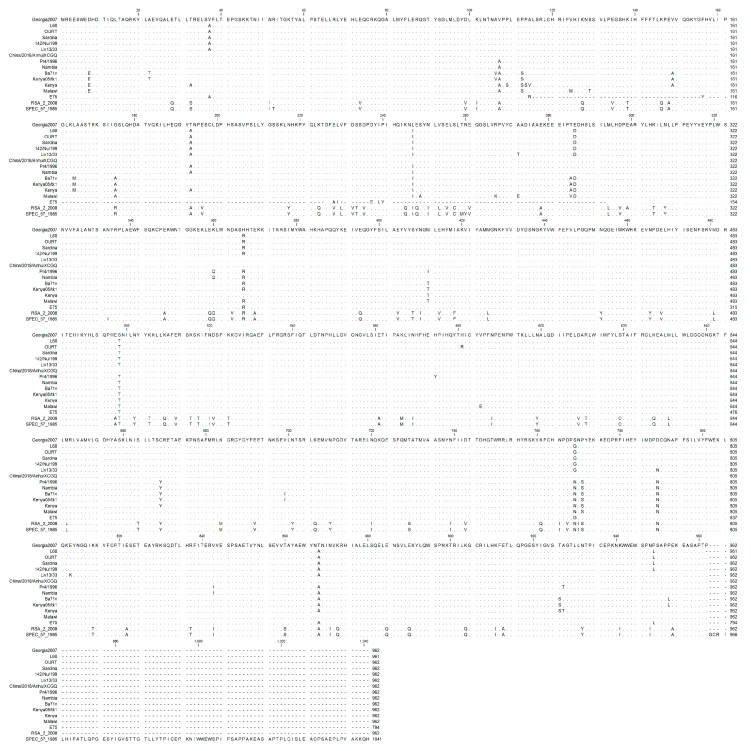
Multiple sequence alignment of the indicated ASFV isolates of viral protein C962R. Matching residues are represented as dots.

Multiple sequence alignments across all published isolates of ASFV were performed using CLC Genomics Workbench, and revealed that most C962R proteins were 962 amino acids in length. However, ASFV isolate E75 had only 794 amino acids, resulting in a deletion between residues 114 and 293 when compared to the ASFV-G isolate; this deletion did not affect any of the identified functional domains identified (Figure 1B and Figure 2). Excluding the E75 isolate from analysis, 821 of 962 residues were conserved in all other isolates. Two recent South Africa isolates, RSA_2_2008 and SPEC_57_1985, were the least similar to ASFV-G. 

The transcriptional activity of the *C962R* gene during the infectious cycle was analyzed using deposited microarray data from a previous study [27]; transcription of *C962R* was first detected at 6 hpi, then throughout the remaining duration of infection with expression kinetics similar to ASFV late protein p72 [4,13], Therefore, *C962R* is expressed late during the virus replication cycle. 

### 3.2. Development of the ASFV-G-ΔC962R Deletion Mutant

**Figure 3:**
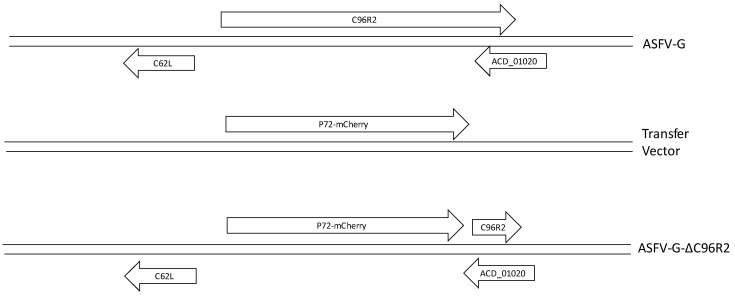
Schematic for the development of ASFV-G-ΔC962R. The transfer vector contains the p72 promoter and a mCherry cassette; the flanking left and right arms are indicated and were designed to have flanking ends to both sides of the deletion/insertion cassette. The resulting ASFV-G-ΔC962R virus with the cassette inserted is shown on the bottom.

To study the function of the *C962R* gene during ASFV replication in cell cultures and the process of virulence in swine, a recombinant virus lacking the *C962R* gene was developed (ASFV-G-ΔC962R). Deletion of *C962R* was achieved by substituting 942 amino acid residues of the *C962R* ORF with a p72mCherry cassette following standard methodologies based on homologous recombination [12]. ASFV-G-ΔC962R was constructed from the highly virulent ASFV Georgia 2007 isolate (ASFV-G). A region spanning 2824-bp (between nucleotide positions 89,075–91,898) was deleted from the ASFV-G genome and substituted with a 1226-bp cassette containing the p72mCherry construct (see Materials and Methods). The obtained recombinant virus contains a deletion in the *C962R* ORF (Figure 3), leaving the C-terminal 65-bp. The remaining 65-bp were left to not disturb the potential ORF ASFV-G-AVD-01020, which is transcribed in the opposite direction on the reverse strand of the ASFV genome. ASFV-G-ΔC962R was obtained after a process of successive limiting dilution purifications in swine macrophage cell cultures. The virus obtained from the last purification round was further amplified in primary swine macrophage cell cultures to obtain a virus stock.

The accuracy of the genetic modifications introduced in ASFV-G-ΔC962R, as well as the conservation of the integrity of the remaining virus genome, was assessed by full a genome sequence obtained by NGS on an Illumina NextSeq^®^ 500. The comparison between ASFV-G-ΔC962R and ASFV-G full length genomes demonstrated a deletion of 2824 nucleotides that is consistent with the introduced modifications. In addition, the ASFV-G-ΔC962R genome contained a 1294 nucleotide insertion corresponding to the introduction of the p72-mCherry cassette sequence. No additional significant differences were observed between the ASFV-G-ΔC962R and ASFV-G genomes, confirming that the ASFV-G-ΔC962R virus did not acquire additional mutations during the process of homologous recombination or limiting dilution purification procedure. NGS results also demonstrated the absence of any residual parental ASFV-G genome as a potential contaminant of the ASFV-G-ΔC962R stock.

### 3.3. Replication of ASFV-G-ΔC962R in Primary Swine Macrophages

**Figure 4:**
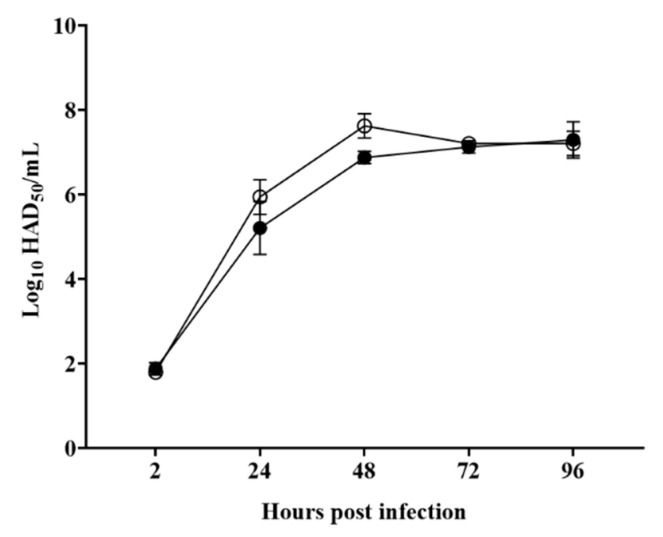
In vitro growth kinetics in primary swine macrophage cell cultures for ASFV-G-ΔC962R and parental ASFV-G, infected (MOI = 0.01) with ASFV-G-ΔC962R or parental ASFV-G viruses. Samples were taken from three independent experiments at the indicated time points and titrated. Data represent means and standard deviations. Sensitivity using this methodology for detecting virus: >log10 1.8 HAD_50_/mL. No significant differences in viral yields between viruses were observed at any timepoint which had been tested determined using the Holm–Sidak method (α = 0.05), without assuming a consistent standard deviation. All calculations were conducted using the software GraphPad Prism version 8.

Based on its predicted NTPase activity, deletion of the *C962R* gene could have an effect on the virus replication cycle. To assess its function during virus replication, the in vitro growth kinetics of ASFV-G-ΔC962R were evaluated in swine macrophage cultures. ASFV-G-ΔC962R kinetics were compared to that of the parental ASFV-G in a multistep growth curve. Macrophage cultures were infected with either virus at a MOI of 0.01, and samples were collected at 2, 24, 48, 72 and 96 hpi. Results demonstrated that ASFV-G-ΔC962R displayed a similar growth kinetic when compared to the parental ASFV-G (Figure 4). Thus, deletion of the *C962R* gene does not significantly affect the ability of ASFV-G to replicate in swine macrophages. This was surprising, considering the *C962R* gene encodes for an NTPase which share similarities with D5 DNA primase of vaccinia virus, a gene involved in the initiation of DNA replication [28]. However, it is possible that C962R function can be replaced by the role of another ASFV gene that also has potential NTPase and helicase activity, such as gene F1055L.

### 3.4. Assessment of ASFV C962R Virulence in Swine

**Figure 5:**
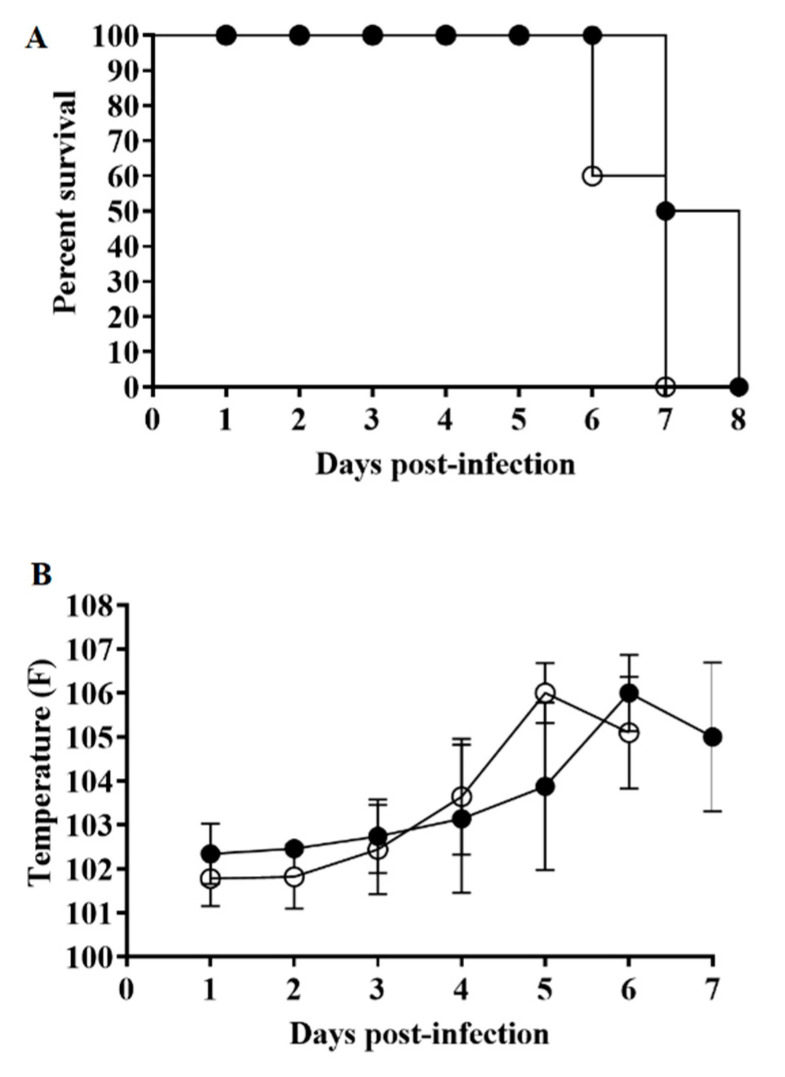
Evolution of mortality (**A**) and body temperature (**B**) in animals (5 animals/group) Intramuscular (IM) infected with 10^2^ HAD_50_ of either ASFV-G-ΔC962R (filled symbols) or parental ASFV-G (open symbols). No significant differences in rectal temperatures between both groups of pigs were found at any sample time tested using the Holm–Sidak method (α = 0.05) without assuming a consistent standard deviation. All calculations were conducted using the software GraphPad Prism version 8.

To assess the consequences of the deletion of the *C962R* gene in ASFV-G virulence, a group (*n* = 5) of 80–90-pound pigs were IM inoculated with 10^2^ HAD_50_ per animal, while an additional control group was also IM inoculated with 10^2^ HAD_50_ of the parental ASFV-G. As expected, animals inoculated with virulent ASFV-G exhibited an increase in body temperature (>104 °F) by day 4–5 pi that was followed by the quick appearance of ASF-associated clinical signs (including anorexia, depression, purple skin discoloration, staggering gait and diarrhea) (Table 1 and Figure 5).

Signs of the disease rapidly aggravated, and animals were euthanized in extremis by day 5–6 pi. Animals receiving ASFV-G-ΔC962R presented with a clinical disease very similar to that described for animals infected with ASFV-G. Both the time of presentation and severity of the clinical signs resembled those present in animals inoculated with the parental virus. Consequently, deletion of the *C962R* gene does not alter the virulence phenotype of the highly virulent ASFV-G isolate.

**Figure 6:**
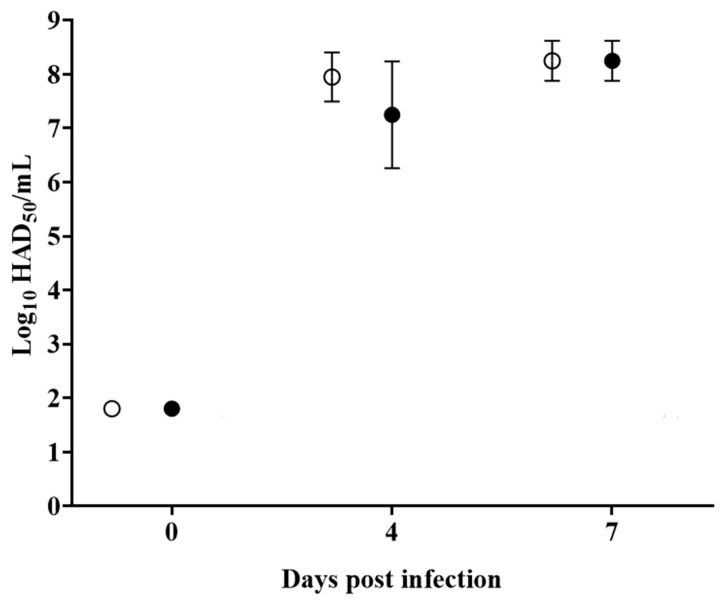
Viremia titers detected in pigs IM inoculated with 10^2^ HAD_50_ of either ASFV-G-ΔC962R (filled symbols), or ASFV-G (empty symbols). Each symbol represents the average of animal titers in each of the groups. Sensitivity of virus detection: > log10 1.8 TCID_50_/_mL_. No significant differences in viremia values between both groups of pigs were found at any sample time tested using the Holm–Sidak method (α = 0.05) without assuming a consistent standard deviation. All calculations were conducted on the software GraphPad Prism version 8.

The analysis of viremias in animals IM infected with parental ASFV-G demonstrated expected high titers (10^6^–10^7.85^ HAD_50_/mL) on day 4 pi, remaining similarly high by day 7 pi, when all animals were euthanized. Animals inoculated with ASFV-G-ΔC962R presented with viremia values ranging from 10^6^–10^6.8^ HAD_50_/mL by day 4 pi, reaching similar titers of those animals infected with ASFV-G by day 7 pi, which was also the last sample time taken before animals were humanely euthanized (Figure 6). The presentation of these indistinguishable clinical signs suggests that the *C962R* gene is not required for ASFV virulence.

In summary, we determined that C962R, a highly conserved protein among ASFV isolates that is similar to the iridovirus primase family fused to D5-like helicase domains [20], is an early protein that is a nonessential gene, since its deletion from the ASFV-G genome does not significantly alter virus replication in swine macrophage cultures, and importantly, is not critical for ASFV virulence in swine, as the deletion mutant ASFV-G-ΔC962R had similar pathogenesis as the parental ASFV-G.

## Figures and Tables

**Table 1:** Swine survival and fever response following infection with ASFV-G-ΔC962R and parental ASFV-G.

			Fever		
Virus (10^2^ HAD_50_)	No. of Survivors/Total	Mean Time to Death(± SD)	No. of Days to Onset (± SD)	DurationNo. of Days (± SD)	Maximum Daily Temp, °F (± SD)
ASFV-G-ΔC962R	0/5	5.4 (0.55)	5.4 (0.55)	1.4 (0.55)	106 (0.87)
ASFV-G	0/5	5.2 (0.45)	4.4 (0.55)	1 (1)	106 (0.69)
